# A comparative study of nano-fillers to improve toughness and modulus of polymer-derived ceramics

**DOI:** 10.1038/s41598-021-82365-3

**Published:** 2021-03-26

**Authors:** Mohammad Mirkhalaf, Hamidreza Yazdani Sarvestani, Qi Yang, Michael B. Jakubinek, Behnam Ashrafi

**Affiliations:** 1 Aerospace Manufacturing Technology Center, National Research Council Canada, 5145 Decelles Avenue, Montreal, QC H3T 2B2 Canada; 2 Aerospace Manufacturing Technology Center, National Research Council Canada, 1200 Montreal Rd., Ottawa, ON K1A 0R6 Canada; 3 Division of Emerging Technologies, National Research Council Canada, 100 Sussex Dr., Ottawa, ON K1A 0R6 Canada; 4 Present Address: School of Biomedical Engineering, The University of Sydney, Sydney, NSW 2008 Australia

**Keywords:** Aerospace engineering, Mechanical engineering, Ceramics, Composites, Mechanical properties

## Abstract

Brittleness is a major limitation of polymer-derived ceramics (PDCs). Different concentrations of three nanofillers (carbon nanotubes, Si_3_N_4_ and Al_2_O_3_ nanoparticles) were evaluated to improve both toughness and modulus of a commercial polysilazane (PSZ) PDC. The PSZs were thermally cross-linked and pyrolyzed under isostatic pressure in nitrogen. A combination of mechanical, chemical, density, and microscopy characterizations was used to determine the effects of these fillers. Si_3_N_4_ and Al_2_O_3_ nanoparticles (that were found to be active fillers) were more effective than nanotubes and improved the elastic modulus, hardness, and fracture toughness (*J*_*IC*_) of the PDC by ~ 1.5 ×, ~ 3 ×, and ~ 2.5 ×, respectively. Nanotubes were also effective in maintaining the integrity of the samples during pyrolysis. The modulus and hardness of PDCs correlated positively with their apparent density; this can provide a fast way to assess future PDCs. The improvement in fracture toughness was attributed to crack deflection and bridging observed in the micro-indentation cracks in the modified PDCs. The specific toughness of the modified PDCs was 4 × higher than that of high-purity alumina, and its specific modulus reached that of commercially available technical ceramics. These PDCs can also easily take different shapes and therefore are of interest in protective armor, propulsion, thermal protection, device packaging and biomaterial systems.

## Introduction

Ceramics are technologically important materials due to their high strength, low density, excellent thermal stability, and high oxidation/corrosion resistance. As such, they are important in structural applications involving high service temperatures; e.g., in engine components, high temperature furnaces and exhausts^1–4^. However, in comparison to high performance alloys, ceramics offer limited shape flexibility in manufacturing and suffer from low fracture toughness and susceptibility to thermal shocks^5^. In traditional ceramic manufacturing processes^6,7^, porosity usually increases with the freeform capability of the manufacturing process. Polymer-derived ceramics (PDCs) can address this issue. PDCs are produced in a two-stage process where, in the first stage, the polymer is covalently cross-linked (cured) to a green state. In the second stage (pyrolysis), the temperature is increased (usually to more than 800 °C) so that the organic moieties (e.g., methyl groups attached to the Si atoms in a polysilazane, PSZ) are eliminated and a ceramic residue called a PDC is obtained^8–14^. Since the ceramic is derived from a polymer, the processing method has several advantages over more traditional methods. These advantages include ease of fabrication, near-net shape manufacturing of complex shapes (e.g., for advanced cooling systems in gas turbine engines), and being relatively environmental friendly^8,15,16^. However, the main drawback of PDCs is the significant gas release and shrinkage during the transformation of the cross-linked polymer to the ceramic^17^. This gas release induces porosity in the resulting material^9,18^. To decrease the porosity of PDCs, two main approaches have been employed^4,5^: (1) addition of nanometer sized fillers^9,10,12,19–21^ and (2) using pressure during pyrolysis^11^. Passive fillers dilute the preceramic polymer and therefore decrease the amount of gas generated and the associated volume shrinkage. This can reduce the possibility of forming cracks/voids during pyrolysis^12,20^. On the other hand, active fillers can react with the decomposition gases generated during pyrolysis to form a secondary phase (e.g., silicon oxynitride)^10,11,22,23^, which reduces the amount of shrinkage and void formation^10,24^. Pressurizing the polymer during pyrolysis can also decrease the void content due to pressure-driven flow of the material into the cavities resulting from the gas release. Here we present a comparative study of different fillers (Al_2_O_3_ and Si_3_N_4_ nanoparticles as well as carbon nanotubes) added to a commercial PSZ resin on the properties of the resulting PDCs, which are characterized in terms of composition, density, harness, modulus, and fracture toughness to guide the selection of these fillers for future studies (e.g. on 3D–4D printing of PDCs).

## Results and discussion

**Figure 1:**
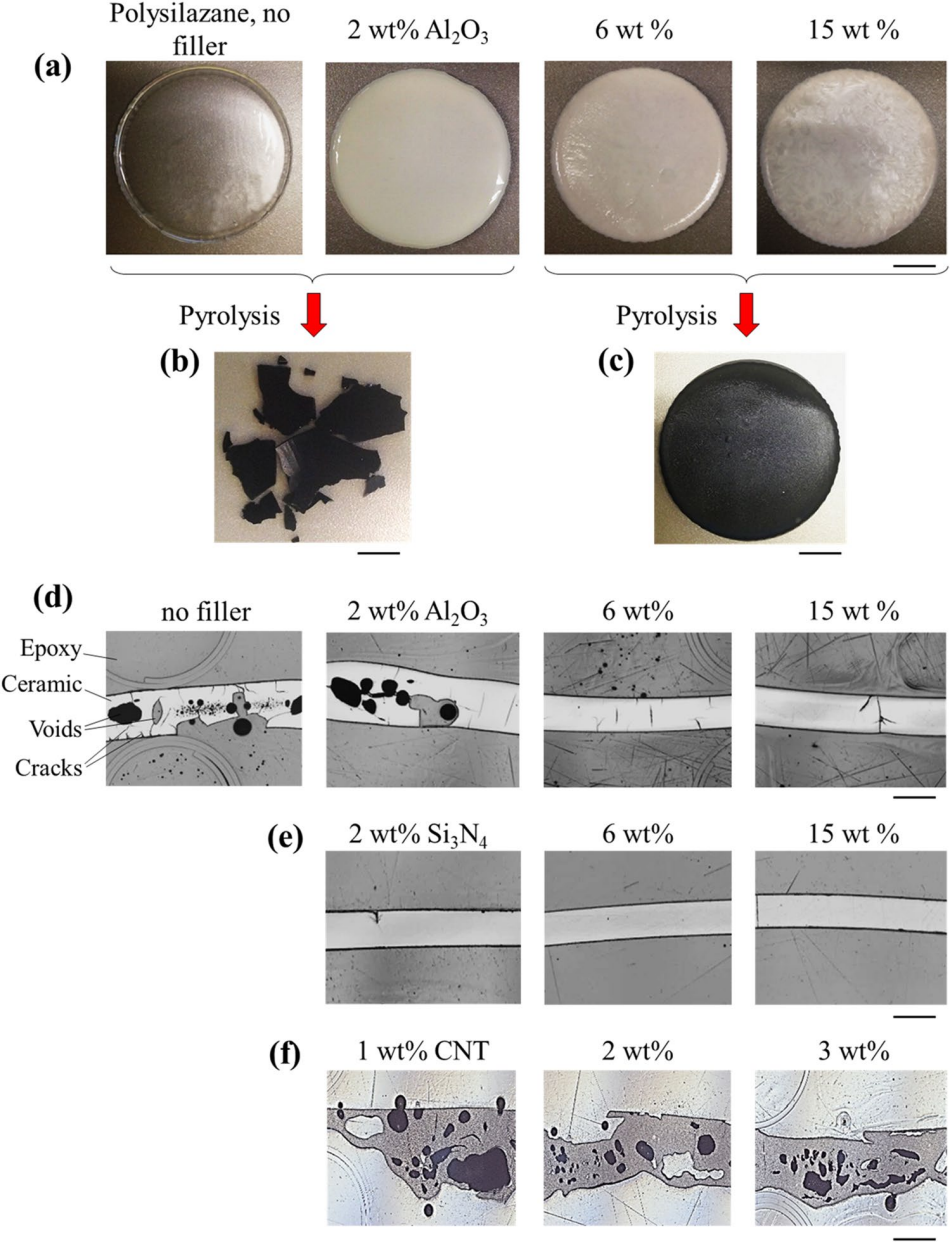
Nano-modified preceramic polymers. (**a**) Optical images of samples with different concentrations of alumina nano-fillers before the pyrolysis, and (**b**,**c**) After the pyrolysis. (**d**–**f**) Optical microscopy images of PDCs with different concentrations of (**d**) Al_2_O_3_, (**e**) Si_3_N_4_, and (**f**) CNTs. Scale bars are (**a–c**) 15 mm, (**d–f**) 2 mm.

Samples with different concentrations of fillers were easily detached from the aluminum mold after crosslinking to the green state (at 150 °C for 15 min) in the presence of a radical initiator (see “Materials and methods” section, Fig. 1a). Filler concentrations in the range of 0–15 wt% were explored for alumina and silicon nitride nanoparticles. Concentrations beyond 15 wt% resulted in a very viscous paste that could not be processed. For nanotubes, this threshold concentration was 3 wt%. After pyrolysis (at 1000 °C under isostatic pressure of 30 MPa in nitrogen), extensive macro-cracking and high volume of voids were observed within the material with no or low concentration (i.e., 2 wt%) of alumina (Fig. 1a,b). Tailoring the heating and cooling cycles did not prevent shattering of the samples or creation of voids. However, higher concentrations of alumina or silicon nitride eliminated the cracking and resulted in solid samples with relatively retained shape (Fig. 1c). At low filler concentrations, most voids were located halfway through the thickness of the samples (Fig. 1d), which provided a sign that they resulted from pyrolysis reaction gases that could not escape from the material. Although there were fewer voids with a higher concentration of alumina nano-filler (6 and 15 wt%, Fig. 1d), there were some macroscale-cracks in the material. Conversely, samples containing silicon nitride nano-fillers were almost free of defects in the optical microscope images (Fig. 1e). In contrast to the alumina and silicon nitride nano-particles, adding CNTs did not dramatically decrease the void content (Fig. 1f), although the integrity of the samples was retained during pyrolysis by adding CNTs, an effect that has been observed in previous studies^25^.

**Figure 2:**
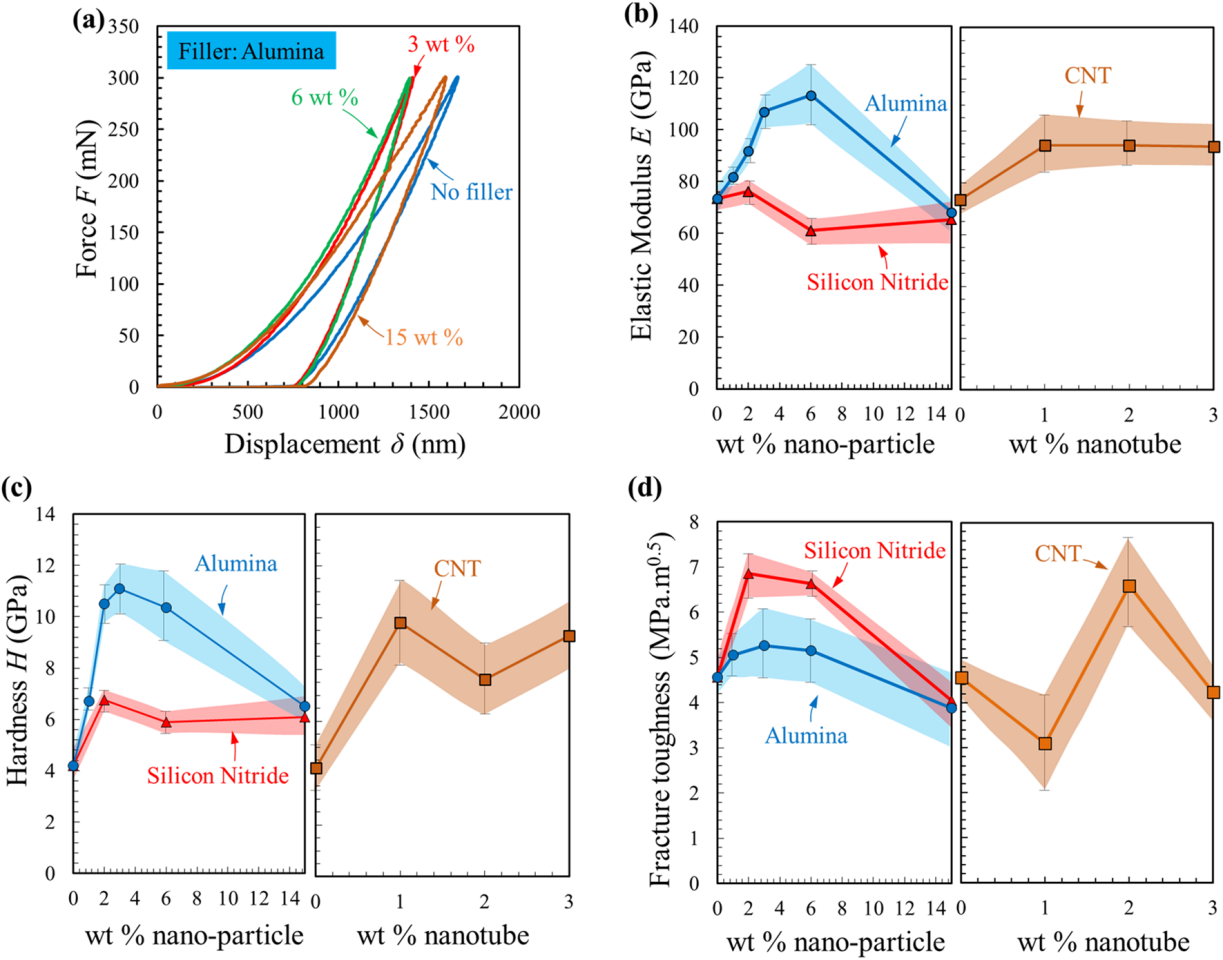
Properties of the modified PDCs obtained from nano/micro-indentation. (**a**) Representative nano-indentation force–displacement results for the samples containing alumina nano-filler. (**b**) Elastic Modulus, (**c**) hardness and (**d**) fracture toughness of the PDC modified by different concentrations of alumina, silicon nitride or carbon nanotube nano-fillers. Colored regions show 2 standard deviations (number of samples *N* = 6).

Figure 2 shows the mechanical test results obtained from nano-indentation and micro-indentation fracture with different concentrations of alumina (1, 3, 6, and 15 wt%) and silicon nitride (2, 6, and 15 wt%) nanoparticles as well as different concentrations of CNTs (1, 2, and 3 wt%). Indentation was selected over other common mechanical tests, such as 3-point bending (for measuring the modulus) and single edge notch test (for measuring the fracture toughness), because of: (a) the irregular shape of the ceramic samples, in particular the baseline PSZ-derived ceramic, which shattered into smaller pieces during pyrolysis, and (b) the high porosity of the samples with low filler content, which resulted in their breakage at an unintended position during the bending tests. Several studies show that the fracture toughness values obtained from indentation tests on ceramics agree with those obtained from 3-point bending notch or double cantilever beam fracture tests^26,27^. Also, indentation has been used in the literature to measure both elastic modulus and toughness of PDCs^28–30^. However, the indentation fracture toughness values of PDCs might not be absolute due to possible densification of the samples under the indenter. The fracture toughness values are therefore best-used as a comparison between qualitatively similar materials^30^, such as those in the present work.

Nano-indentation experiments (maximum force: 300 mN) were used to characterize the hardness and elastic modulus of the material. Micro-indentation tests (max. force: 49 N) were used to create cracks within the ceramics and measure their fracture toughness^26,31^ (see “Materials and methods” section for details); creating cracks was not possible using nano-indentation. All measurements were performed on the polished surfaces of the samples embedded in epoxy. The overall shape of the indentation force–deflection curves (loading and unloading) was similar across all the samples (Fig. 2a). The slope of the indentation unloading curve that shows the elastic recovery of the material initially increased with filler content (up to 6 wt% for alumina) and then decreased for higher concentrations. Figure 2b to 2d respectively show how the elastic modulus, hardness and fracture toughness of PDCs change with concentration of nano-fillers. The elastic modulus of the PDC without filler was found to be ~ 73 GPa, which was identical to the values An et al. obtained using the same materials and processing parameters^12^. Shah and Raj reported higher values for the modulus^32^, which could be due to their different processing where PSZ was molded under isostatic pressure during the cross-linking stage (green stage).

The elastic modulus of the material increased to ~ 113 GPa (55% improvement) by addition of 6 wt% of alumina nano-powder to the resin, while additional alumina resulted in a degradation of the modulus (Fig. 2b) likely due to the clustering of nanoparticles, also observed in other PDCs^33^. Similar trends were observed for the hardness (Fig. 2c) and fracture toughness (Fig. 2d) of the material; however, only 15% improvement in fracture toughness could be obtained with Al_2_O_3_ nano-fillers.

Compared to Al_2_O_3_ nano-fillers, the results obtained with silicon nitride showed smaller improvements in terms of modulus and hardness: addition of the Si_3_N_4_ nano-filler yielded only 4% and 50% improvements in the modulus (Fig. 2b) and hardness, respectively (Fig. 2c). However, fracture toughness values were significantly improved by adding silicon nitride nanoparticles (Fig. 2d). While the same load and indenter were used for all the samples, the scanning electron microscopy (SEM, Zeiss, Germany) images showed that the micro-indented crack length *c* was significantly decreased for the samples modified with a small amount (1–6%) of either alumina or silicon nitride nanoparticles (Supplemental information, Fig. S1). The fracture toughness (in terms of stress intensity factor *K*_*IC*_) of the material with optimum concentration (2 wt%) of Si_3_N_4_ nano-filler was ~ 1.5 times higher than that of the baseline (control) ceramic, and reached values as high as *K*_*IC*_ = 7 MPa m^0.5^. Toughness in energy terms (*J*_*IC*_) is expressed as $J_{IC} = {{K_{IC}^{2} } \mathord{\left/ {\vphantom {{K_{IC}^{2} } E}} \right. \kern-\nulldelimiterspace} E}$ and is ~ 0.75 kJ m^−2^ for the PDC with 2 wt% Si_3_N_4_ nano-filler, which is ~ 2.5 × more than baseline PDC (*J*_*IC*_ ≈ 0.29 kJ m^−2^). Further, the fracture toughness of this PDC (i.e., from polysilazane modified by 2 wt% Si_3_N_4_) is twice that of literature reports for high-purity alumina obtained using similar indentation and crack length measurements (~ 3.5 MPa.m^0.5^^27,34^). We also computed the specific toughness (toughness per density) of this material and found it to be 0.0035 MPa.m^0.5^/(kg/m^3^), which is ~ 4 × that of alumina. While the PDCs with 2 wt% Si_3_N_4_ outperformed traditional ceramics in terms of fracture toughness, their specific modulus (0.038 MPa/(kg/m^3^)), was 50% less than that of alumina (0.075 MPa/(kg/m^3^)). For all properties and all the fillers, with the exception of the modulus of alumina-modified PDCs, degradation in properties started at concentrations < 6 wt% filler. We therefore did not explore concentrations in the 6 wt% to 15 wt% region in more detail. However, the 15 wt% sample served as an extreme case for supporting characterizations (e.g., composition).

**Figure 3:**
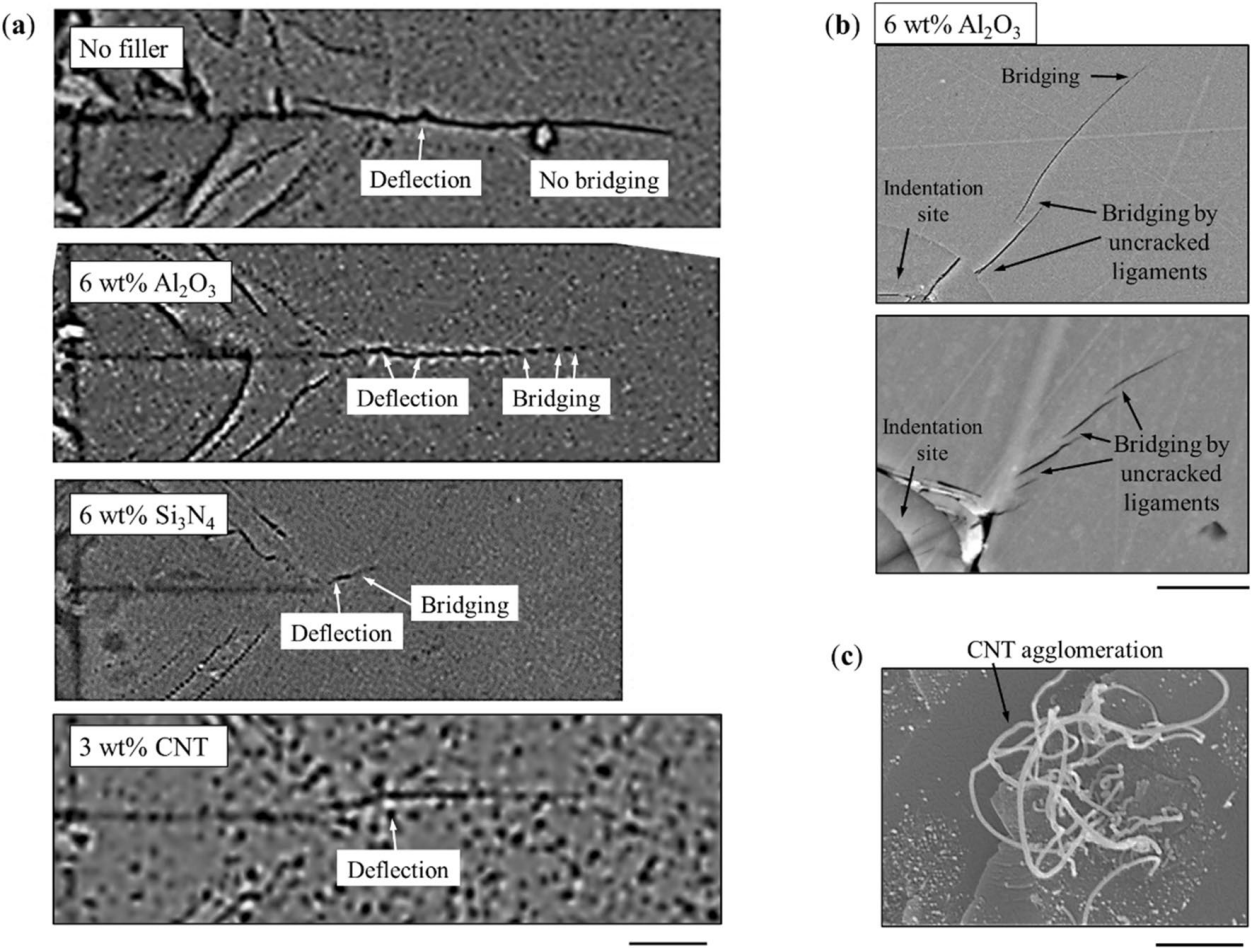
Toughening mechanisms shown on representative SEM micrographs of micro-indentation cracks. (**a**) Comparison between crack propagation in **s**amples are with no fillers, 6 wt% Al_2_O_3_, 6 wt% Si_3_N_4_, and 3 wt% CNT. (**b**) A closer look at the crack bridging toughening mechanism in the Al_2_O_3_ modified PDCs. (**f**) Nanotube agglomeration observed on the surface of CNT-modified samples. All the cracks were obtained with the same force on PDCs. For the representative images of all the samples, see Fig. S1. Scale bars (**a**) 15 µm; (**b**) 15 µm, and (**c**) 1 µm.

Comparison of the fracture patterns (Fig. 3a) showed that the decreased crack length and consequently increased fracture toughness of PDCs modified by nanoparticles (Al_2_O_3_ and Si_3_N_4_) is due to the crack deflection and bridging. While slight deflection was also observed for the non-modified PDCs, no bridging was observed for these samples. To show the effects of filler on crack deflection, we measured the ratio of the total crack length to the equivalent length of a crack that would go straight into the material. This ratio increased from 1.032 ± 0.009 for the baseline ceramic to 1.095 ± 0.013 for the sample with 6 wt% Si_3_N_4_, demonstrating the effective role of Si_3_N_4_ to deflect the cracks. We found that this ratio for the Al_2_O_3_-modified PDCs was very similar to that of the baseline*.* The higher toughness of the Si3N4-modifed samples therefore partially stems from this higher crack deflection.

Deflection and bridging could occur at the interface between the multiple phases of the modified PDCs resulting from presence and reaction of filler nanoparticles^35^. A closer view of the bridges in the Al_2_O_3_-modifed PDCs, where the bridging was most prominent, are shown in Fig. 3b. We used energy-dispersive X-ray spectroscopy (EDS) elemental mapping around cracks and in bridging regions. The compositional mapping and local spot spectra in bridge regions with sub-micron resolution did not show consistent differences in elemental composition between the bridging regions specifically and the material overall (e.g., Fig. S2). The bridges may have been therefore resulted from crack arrest mechanisms operating at nanometer or sub-nanometer length scales such as partially unreacted nanoparticles resulting in localized stiffness gradient.

These toughening mechanisms are less extensively observed for the samples modified with nanotubes (Fig. 3a), resulting in unclear trends and significant variations (Fig. 2, and Fig. S1) that might have resulted from CNT agglomerations (e.g., Fig. 3c). Despite these large variations, adding nanotubes resulted in overall improved mechanical properties, which may be due to known effects of CNTs in particular microscopic crack bridging observed in several previous studies on CNT-reinforced PDCs^36,37^. The addition of 1 wt% CNTs into the control ceramic resulted in 50% increase in elastic modulus. However, the elastic modulus of the material remained almost constant with further addition of CNTs. Similar trends were observed for hardness (Fig. 2c) and fracture toughness (Fig. 2d); e.g., hardness increased from ~ 4.1 GPa for the sample with no filler to ~ 9.8 GPa (a 2.5 × improvement) for the sample with 1 wt% CNTs, after which it did not increase further.

**Figure 4:**
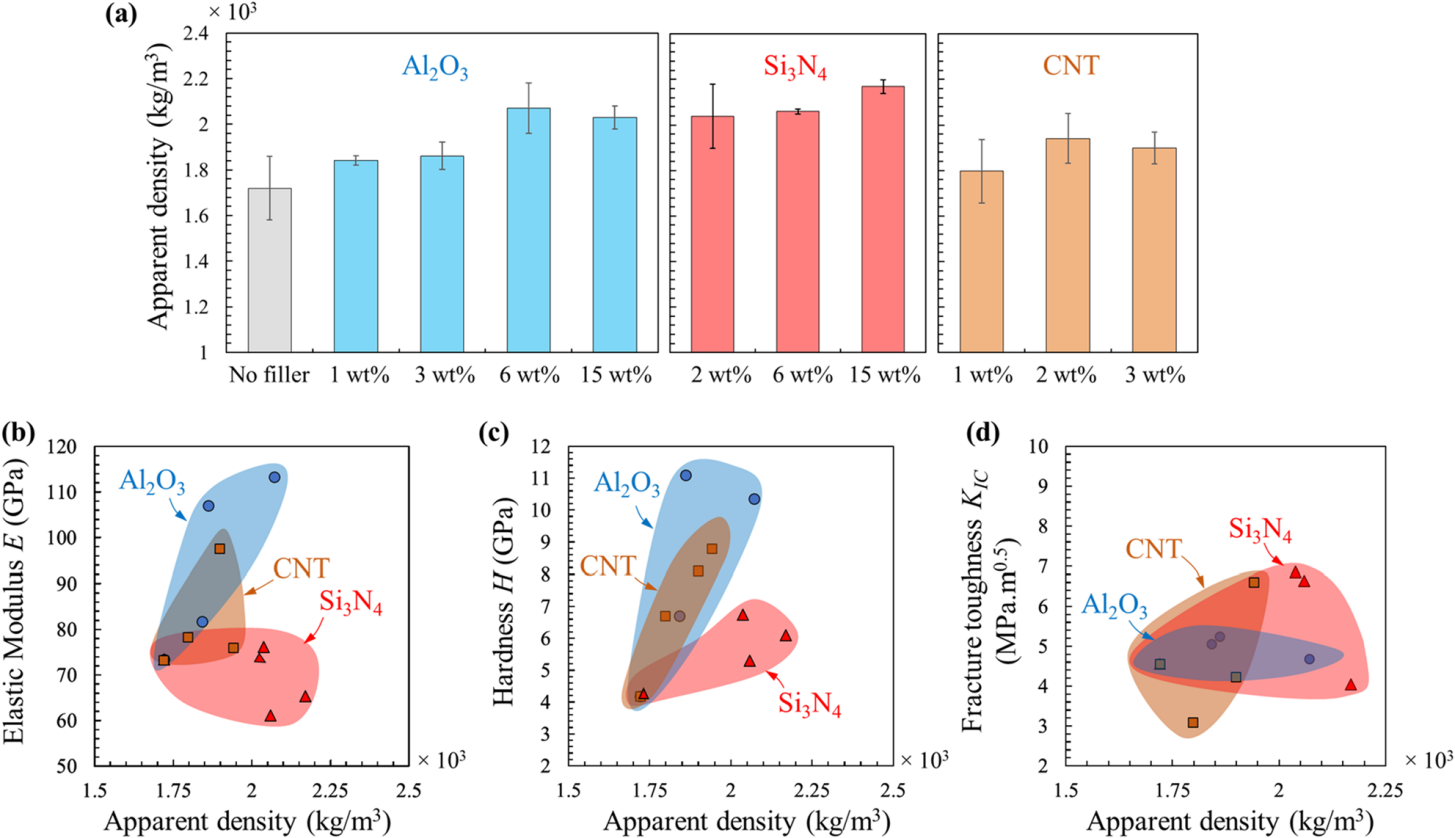
Density–property maps for PDCs modified with nano-particles or CNTs. (**a**) The densities of PDCs made with different concentrations of alumina nanoparticles, silicon nitride nanoparticles, and CNTs. (**b–d**) The correlation between (**b**) modulus, (**c**) hardness, and (**d**) fracture toughness of PDCs and their apparent density.

To explain the trends observed for modulus and hardness, the apparent density of the PDCs was measured (see “Materials and methods” section for details). Like mechanical properties, the apparent density increased with concentration of fillers up to a certain extent after which it did not significantly increase or slightly decreased (Fig. 4a). Plotting the apparent density—property maps revealed that the hardness (Fig. 4b) and modulus (Fig. 4c) correlate positively with the apparent density for most cases. This observation might provide a fast and efficient way to screen and compare other PDCs in future. The fracture toughness did not show a clear correlation with the density (Fig. 4d). The presence of voids might result in competing effects on fracture toughness: it can decrease the load bearing capability of the material, but it might also impede cracks from propagating by blunting them, effectively improving the fracture toughness^38,39^. To validate these finding, we compared our density measurements with the reported values in the literature for the baseline PDC. The solid density of the non-modified PDC was estimated by taking into account the internal void content (measured from the microscopy images) and was found to be 1980 ± 76 kg/m^3^, which is close to the values obtained by An et al.^12^.

**Figure 5:**
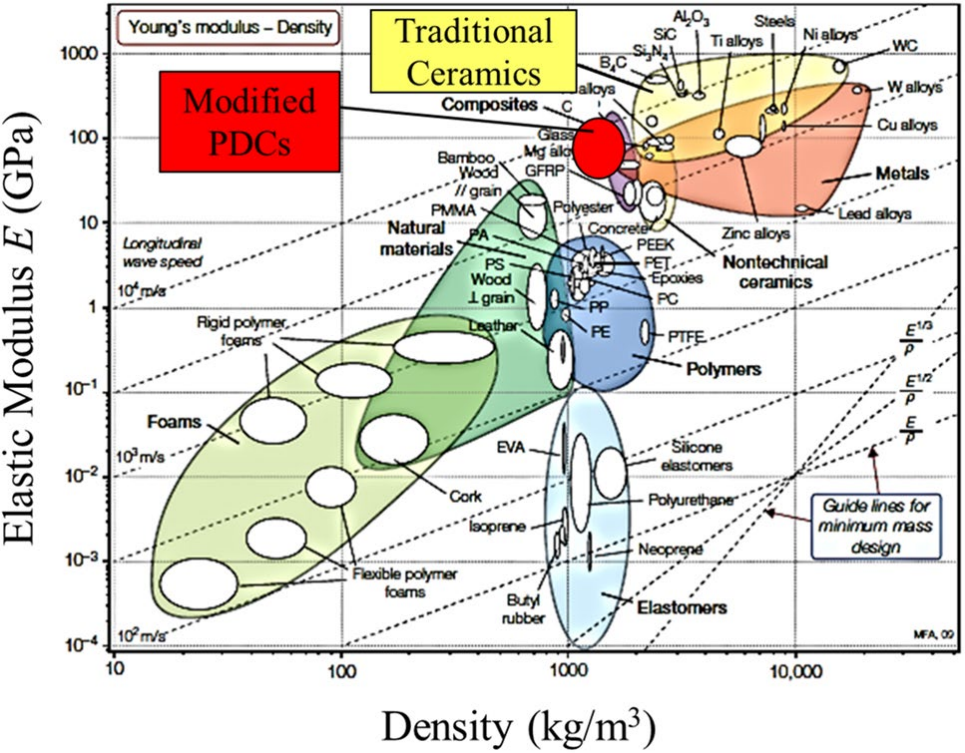
The comparison between mechanical properties of PDCs and other materials. The Ashby charts for modulus—density, showing the properties of PDCs (the chart is adapted from^40^, with permission).

A summary of the modulus and density results can be shown on Ashby chart (Fig. 5). In general, the density and modulus of the PDCs were lower than those of traditional industrial ceramics such as alumina and silicon carbide. Their specific modulus on the other hand is comparable: the present nano-filler modified PDCs and the industrial ceramics lie on the same *E/ρ* guidelines for minimum mass design, while PDCs provide the advantages of being easier to form into complex shapes and compatible with polymer processing methods. Also, the fracture toughness tests show that the modified PDCs can be considerably tougher than commercially available ceramics.

**Figure 6:**
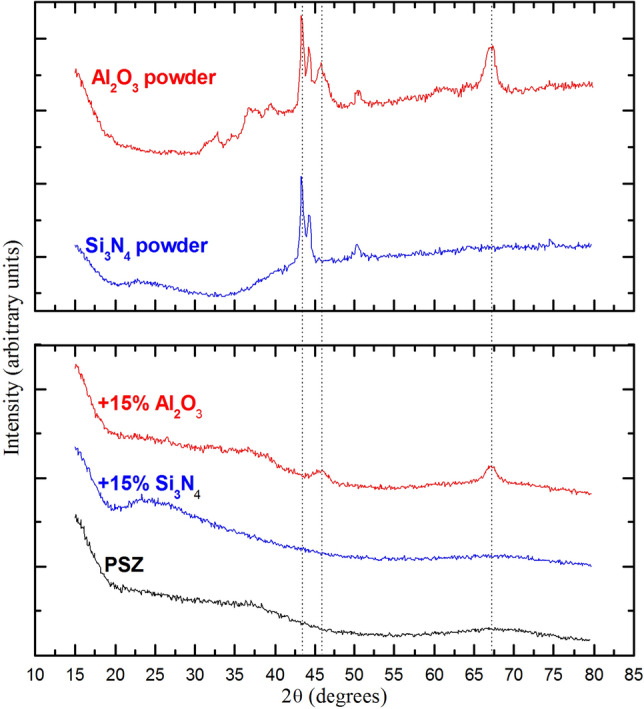
Powder XRD spectra of PSZ-derived ceramic alone and with addition of 15 wt% Al_2_O_3_ or Si_3_N_4_ nanoparticles. Diffraction spectra for the nanoparticles alone were recorded for comparison.

**Figure 7:**
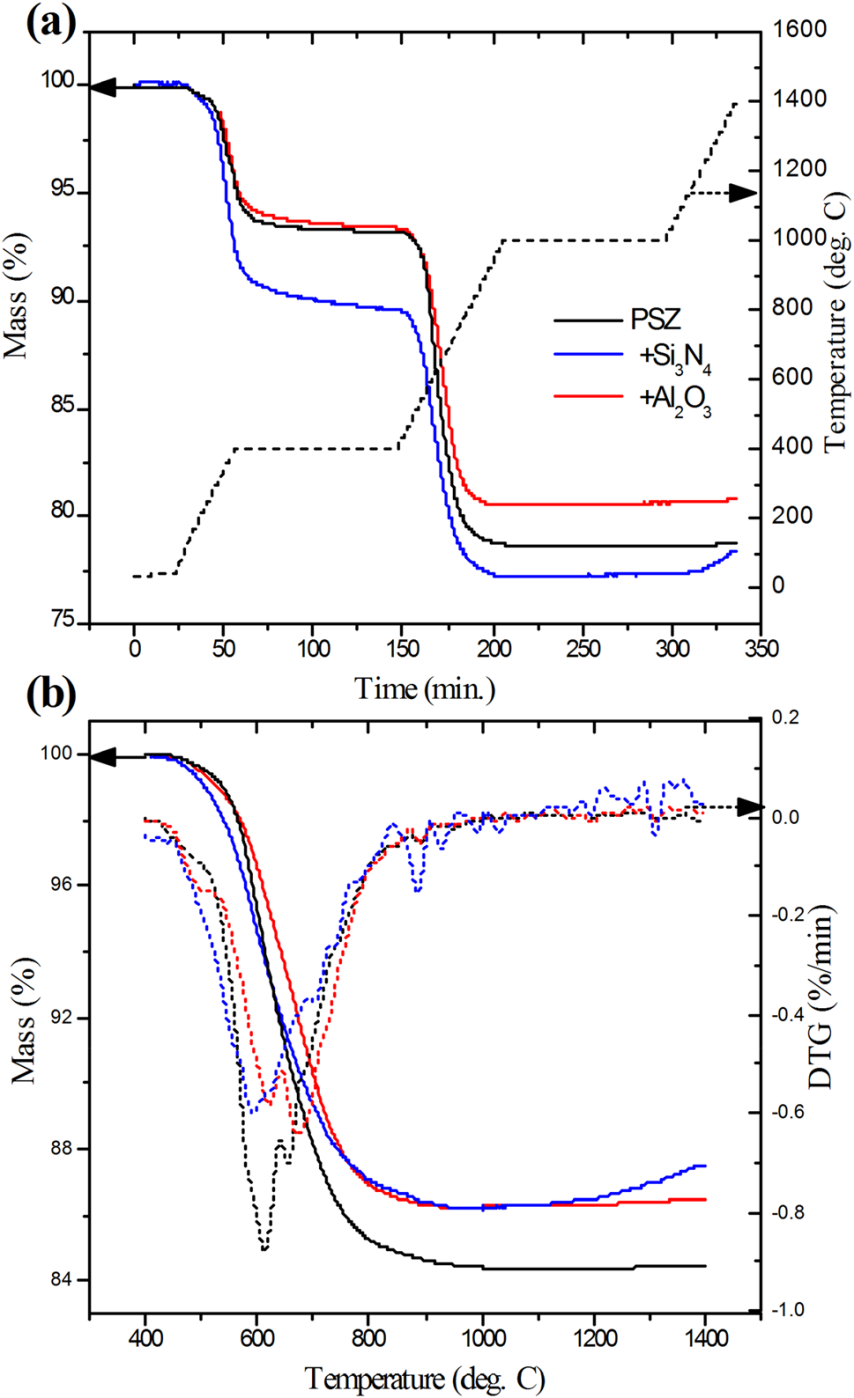
Thermogravimetric analysis under argon. (**a**) The mass loss as a function of time during curing and pyrolysis steps (black dash line indicates the temperature profile) and (**b**) The mass loss during the pyrolysis step only (T > 400 °C) along with the derivative signal (DTG, dotted lines). The DTG signal was smoothed (origin 8 software, FFT filter set at 5 points).

A combination of formation of secondary phases and non-reacted fillers may contribute to the overall density, composition and mechanical properties of final product. To investigate these effects and particularly to reveal whether Al_2_O_3_ and Si_3_N_4_ nanoparticles, which led to the best performance amongst the explored fillers, were active or passive at the pyrolysis temperature (1000 °C), we performed X-ray diffraction analysis (XRD, Fig. 6), X-ray photoelectron spectroscopy (XPS, Table 1), and thermogravimetric analysis coupled to Fourier transform infrared spectroscopy (TGA-FTIR) analysis (Fig. 7 and Figs. S3–S6). XRD was performed on the PDCs modified by 15 wt% Al_2_O_3_ or 15 wt% Si_3_N_4_, as well as for the non-modified PDC, Al_2_O_3_ powder and Si_3_N_4_ powder (Fig. 6). The results show that the non-modified PDC is amorphous, which is consistent with the manufacturer datasheet for pyrolysis at 1000 °C. The XRD spectra for samples made using 15 wt% Si_3_N_4_ did not show the peaks related to Si_3_N_4_ powder. The spectra for samples made using 15 wt% Al_2_O_3_ also showed greater modification of the Al_2_O_3_ peaks than would be expected for dilution alone, supporting the active role of Al_2_O_3_ as well. The low intensity peaks representing Al_2_O_3_ can however demonstrate the presence of the residual alumina. The higher modulus of the Al_2_O_3_-modifed PDCs compared with PDCs modified with other fillers might have therefore resulted from the higher modulus of Alumina (200–450 GPa) compared with SiOCs (50–200 GPa).

**Table 1:** Comparison of the elemental compositions, determined from XPS, for PDCs derived from PSZ 20 with and without Al_2_O_3_, Si_3_N_4_, or CNT nanoparticle fillers.

Resin	Ceramic composition (atomic %)				
	Si	C	N	O	Al
PSZ20	35.6	29.7	20.7	14.0	−
PSZ20 + 1 wt% Al_2_O_3_	36.0	30.0	18.5	15.0	0.5
PSZ20 + 15 wt% Al_2_O_3_	30.8	29.4	17.4	18.0	4.5
PSZ20 + 2 wt% Si_3_N_4_	35.0	29.6	7.9	27.6	−
PSZ20 + 15 wt% Si_3_N_4_	35.3	24.3	7.6	32.8	−
PSZ20 + 3 wt% CNTs	30.5	36.8	14.9	17.8	−

TGA data, following a modified version of the pyrolysis protocol followed for the larger samples (i.e., argon atmosphere; faster ramp rate and shorter holds), shows mass losses associated with the polymerization and pyrolysis (Fig. 7a). The polymerization was largely completed before reaching 400 °C and pyrolysis occurred at temperatures ranging from 400 to 1000 °C. The coupled FTIR spectra (see Supplementary Information, Figs. S3–S6) indicate that hydrocarbon byproducts, which are expected to react with active fillers, were released in the temperature range from 500 to 800 °C. The prominent features of the FTIR spectra in this region are consistent with the spectrum of methane. According to literature^10^, alumina nano-fillers start to react with decomposition gases from the polymer to ceramic conversion at less than 1000 °C and the reaction is complete after four hours at 1000 °C. Heating above 1000 °C, which was possible in the TGA but not in the furnace available for preparation of larger samples, showed no significant mass loss between 1000 and 1400 °C. Mass loss during pyrolysis (Fig. 7b) was lower in samples made with 15 wt% Si_3_N_4_ or Al_2_O_3_ nanoparticle fillers, although the effect was less than would be expected of an entirely passive filler. Pyrolysis mass losses were similar when either 15 wt% Si_3_N_4_ or Al_2_O_3_ was added. However, unexpectedly, the mass loss in curing to the green stage (400 °C) was larger when Si_3_N_4_ was added, suggesting that Si_3_N_4_ may have a different effect than Al_2_O_3_ on the curing of the preceramic polymer. This sample, unlike PSZ alone or with Al_2_O_3_ filler, also did not show indications of water release during the pyrolysis step, which could be a factor in the reduction in voids observed in the microscopy images.

The elemental compositions, determined from XPS (Fig. S7), are shown in Table 1. There is a significant amount of oxygen present despite PSZ being a precursor to SiCN ceramic, which is likely due to the sensitivity of PSZ to moisture and is a complicating factor in the analysis of this material. Addition of 15 wt% Al_2_O_3_ did not dilute the carbon content, which implies that carbon from pyrolysis byproducts was also incorporated as a result of adding the alumina filler (supporting the active filler hypothesis). In the case of Si_3_N_4_, similar inferences can be drawn about the relatively small decrease in carbon content; however, a more dramatic change is observed in the percentages of oxygen (increasing > 2 ×) and nitrogen (decreasing > 2 ×) even for low filler content (2 wt%). This significant change in the ceramic composition could be contributing to the higher toughness of PDCs modified by Si_3_N_4_. The increased oxygen content is explained by the TGA-FTIR observations (see Supplementary Information) where the sample containing Si_3_N_4_, despite being prepared using the same PSZ batch under the same conditions and therefore expected to contain water as well, did not show substantial release of water during the conversion to ceramic. High resolution XPS spectra (Figs. S8, S9) further show a clear shift of the Si(2p) peak in comparison to all samples without Si_3_N_4_. Component fitting shows that this is explained by the silicon environment becoming more Si–O relative to Si–C. The different and high reactivity of the Si_3_N_4_ with polysilazanes observed in this work is likely contributing to the nearly defect-free outcome (Fig. 1). Previous studies also show that the specific volume increases resulted from reaction of active fillers, including Si_3_N_4_, with hydrocarbon by-products and the gas atmosphere (in this case *N*_*2*_) can result in significant reduction of the voids^10,19^.

## Conclusions and outlook

PDCs are a promising class of ceramics due to the ease of forming complex shapes. The mechanical properties of PDCs based on PSZ were improved by adding three different nanoscale fillers (Al_2_O_3_, Si_3_N_4_, and CNTs) and using an isostatic pressure of 30 MPa during pyrolysis. The conclusions are:

- The active fillers, Al_2_O_3_ or Si_3_N_4_ nanoparticles, were more effective than nanotubes in improving mechanical properties: 1.5 ×, 3 × and 2.5 × improvements in modulus, hardness, and the fracture toughness (*J*_*IC*_) were achieved, respectively. The specific modulus of the modified PDCs was similar to technical ceramics, while these PDCs were tougher and much easier to form into complex shapes.
- The change of properties with filler concentration was explained in part by measuring the apparent density of the PDCs, which was found to correlate positively with modulus and hardness of PDCs modified with all the fillers. The apparent density therefore could be used as a very simple and fast comparative tool for modulus and hardness of future PDCs.
- TGA-FTIR, XRD and XPS analysis supported the active role of Al_2_O_3_ or Si_3_N_4_ nanoparticles.

The nanoparticle modified PDCs can lead to improved mechanical performance of different classes of advanced ceramics including 3D printed cellular ceramics and bioceramics. They are promising for fabrication of multiscale (hybrid) ceramic-matrix composites with enhanced mechanical properties and with reduced number of infiltration-pyrolysis cycles required for making dense ceramic-matrix composites. Future studies combining active fillers and nanotubes might result in PDCs that benefit from both the bridging toughening of nanotubes and low porosity of PDCs modified with active fillers. This study can also lead to improving the properties of PDCs made by variety of techniques (e.g. 3D–4D printing) and in turn to expansion of PDC applications to the areas not explored to date (e.g., topologically interlocked ceramics)^41^. Other mechanical (e.g., cyclic, single edge notch, and impact) testing, as well as thermal/chemical stability testing of PDCs at room and elevated temperatures are important components of future study and optimization of this and similar systems.

## Materials and methods

### Materials

A polysilazane (commercial name Ceraset PSZ 20) from KiON industries was used as the preceramic polymer^42^. In the absence of oxygen, the curing time for this polysilazane was extremely long (up to 24 h). By adding 3 wt% dicumyl peroxide (ACROS Organics) as a radical initiator, the curing time was reduced to 15 min in vacuum at 150 °C. Al_2_O_3_ nano-powder (13 nm particle size, Sigma Aldrich 718475), Si_3_N_4_ nano-powder (< 50 nm aspherical particle size, Sigma Aldrich 636703), and CNTs (NC 7000™ industrial grade multi-walled CNTs from Nanocyl, average diameter of 9.5 nm) with different concentrations were employed as nano-fillers. Prior to mixing, the nanoparticles were dried in vacuum at 150 °C for two hours. After cooling to room temperature, the powder was mixed with the polymer resin using a planetary mixer (Thinky ARE-310) at 2000 rpm for 3 min. The degassing of the mixture was done in two stages: in the planetary mixer at 2200 rpm for 3 min, and then under vacuum for 30 min. 10 g of the mixture was then poured into a circular aluminum mold with diameter of 6 cm. The material was subsequently crosslinked at 150 °C for 15 min and then removed from the mold. The resulting samples were heated to 400 °C (2 °C/min) and held at this temperature for four hours to finish the cross-linking of the polymer. Under an isostatic pressure of 30 MPa in nitrogen, the temperature was then increased to 1000 °C (2 °C/min) and held at 1000 °C for four hours for pyrolysis^43^. The samples were then cooled to room temperature at 2 °C/min. The optical microscopy images were taken with an Olympus microscope (Tokyo, Japan).

### Nano/micro-indentation

For nano-indentation, a diamond Berkovich indenter was used and the tests were performed at least on seven different spots using a nano hardness tester (CSM, Australia). Displacement controlled testing (1000 nm/min) was used and the tests were continued up to the 300 μN load. In all cases, the peak indentation load was held for 15 s to account for the creep behavior of the indented material. The Oliver and Pharr method^44^ was used to analyze the data. The slope of the unloading curve *S* at the initial stages of unloading was calculated. The effective contact modulus $E_{eff}$ was then calculated as:

1 $$E_{eff} = \frac{{\sqrt {{\pi \mathord{\left/ {\vphantom {\pi A}} \right. \kern-\nulldelimiterspace} A}} }}{2\beta }S,$$

where *A* is the area function (the indenter shape function) of the Berkovich indenter, and $\beta$ is a correction factor to account for the deviation in stiffness caused by lack of symmetry of the Berkovich tip; $\beta$ is set to 0.77 following^44^. The area function of the indenter tip was calibrated for an accurate projected area (*A*) as a function of contact depth (*h*_*c*_). This was accomplished by performing 110 indentation tests at 22 different loads on a reference material (well-polished fused silica; modulus *E* = 72.0 GPa, Poisson’s ratio ʋ = 0.17). The elastic modulus (*E*) of the ceramic was then calculated using:

2 $$\frac{1}{{E_{eff} }} = \frac{{1 - \nu^{2} }}{E} + \frac{{1 - \nu_{i}^{2} }}{{E_{i} }},$$

where *E*_*i*_ is the modulus of the indenter, *ʋ* is the Poisson’s ratio of the ceramic and *ʋ*_*i*_ is the Poisson’s ratio of the indenter. The Poisson’s ratios were assumed to be 0.2 (a typical value for ceramics^45^) and *E*_*i*_ is 1050 GPa for the diamond indenter.

The fracture toughness values were measured by indentation micro-fracture experiments using a direct crack length measurement technique^26^. The samples were loaded at a fixed peak force of 5 kgf (49.05 N) at a rate of 600 mN/min using the same hardness tester as used for the nanoindentation experiments. The indenter was then retrieved at the rate of 600 mN/min. Several formulations have been developed to estimate the fracture toughness of hard and brittle material using this technique. One of the most accepted methodologies (Antis et al.^26^) was employed, wherein the fracture toughness (i.e., *K*_*IC*_) is stated as:

3 $$K_{IC} = A\left( \frac{E}{H} \right)^{n} \frac{P}{{c^{3/2} }},$$

where *E*, *H*, *P* and *c* are elastic modulus of the material, hardness, peak load and characteristic dimensions of radial/median cracks, respectively. The constants *A* = 0.016 and *n* = 0.5 have been empirically determined by Antis et al.^26^.

### Chemical, compositional and density characterization

To gain an understanding of the nature of the chemical reactions during pyrolysis (e.g., passive or active role of the fillers), and to measure changes in the density of the material by adding fillers, we used a combination of TGA-FTIR, SEM–EDS, XRD, XPS, and sink-float density measurements. TGA (Netzsch STG 449 F1; 10 °C/min heating rate) coupled to an FTIR spectrometer (Bruker Tensor 27) was used to measure mass as a function of temperature during heating/pyrolysis under argon and simultaneously record the IR spectra of the species associated with the mass loss. Polished, indented samples were imaged by SEM (Hitachi SU3500) with an Oxford Instruments X-act EDS detector. Samples were imaged uncoated in backscattered electron mode. XRD spectra were measured using a Bruker AXS D8 Advance X-ray diffractometer. Elemental compositions were determined from XPS survey scans (Kratos AXIS Ultra DLD spectrometer with a monochromated Al K-alpha beam (1489.6 eV)) using relative sensitivity factors referenced to carbon. XPS measurements were performed under high vacuum (5 × 10^–9^ Torr) and three spots (300 × 700 μm area) were measured on each sample to verify consistent compositional results for each sample. PDC sample for both XRD and XPS were first ground to powders using a mortar and pestle. The density of the samples was measured using a sink-float analysis and liquid density measurements. The analysis was performed by placing three small pieces (a few millimeters in dimensions) of the ceramic sample in a heavy liquid (aqueous sodium polytungstate solution) at 25 °C. The density of the liquid was adjusted by adding water or concentrated sodium polytungstate solution until the samples were neutrally buoyant. At this point, the densities of the solid sample and the liquid were equal and the liquid density was determined using an automatic density meter (Rudolph Research).

## Supplementary Information

Supplementary Information
