# Supplementary material for: A comparative study of nano-fillers to improve toughness and modulus of polymer-derived ceramics
Source: Sci Rep. 2021 Mar 26;11:6951. doi: 10.1038/s41598-021-82365-3 (PMC7997883; doi:10.1038/s41598-021-82365-3)
Supplement: Supplementary file 1 — Supplementary Information [file 41598_2021_82365_MOESM1_ESM.docx]

**Supplementary Information**

**A comparative study of nano-fillers to improve toughness and modulus of polymer-derived ceramics**

M. Mirkhalaf^1*†^, H. Yazdani Sarvestani^1^, Q. Yang^2^, M.B. Jakubinek^3^, B. Ashrafi^1*^

*^1^National Research Council Canada, Aerospace Manufacturing Technology Center, 5145 Decelles Avenue, Montreal, QC H3T 2B2, Canada*

*^2^National Research Council Canada, Structures, Materials and Manufacturing Laboratory, 1200 Montreal Rd., Ottawa, ON K1A 0R6, Canada*

*^3^National Research Council Canada, Division of Emerging Technologies, 100 Sussex Dr., Ottawa, ON K1A 0R6, Canada*

**Email:* mohammad.mirkhalaf@sydney.edu.au, and behnam.ashrafi@nrc-cnrc.gc.ca

^†^Present address: School of Mechanical and Aerospace Engineering, The University of Sydney, NSW Australia 2008

**SEM images for indentation measurements**


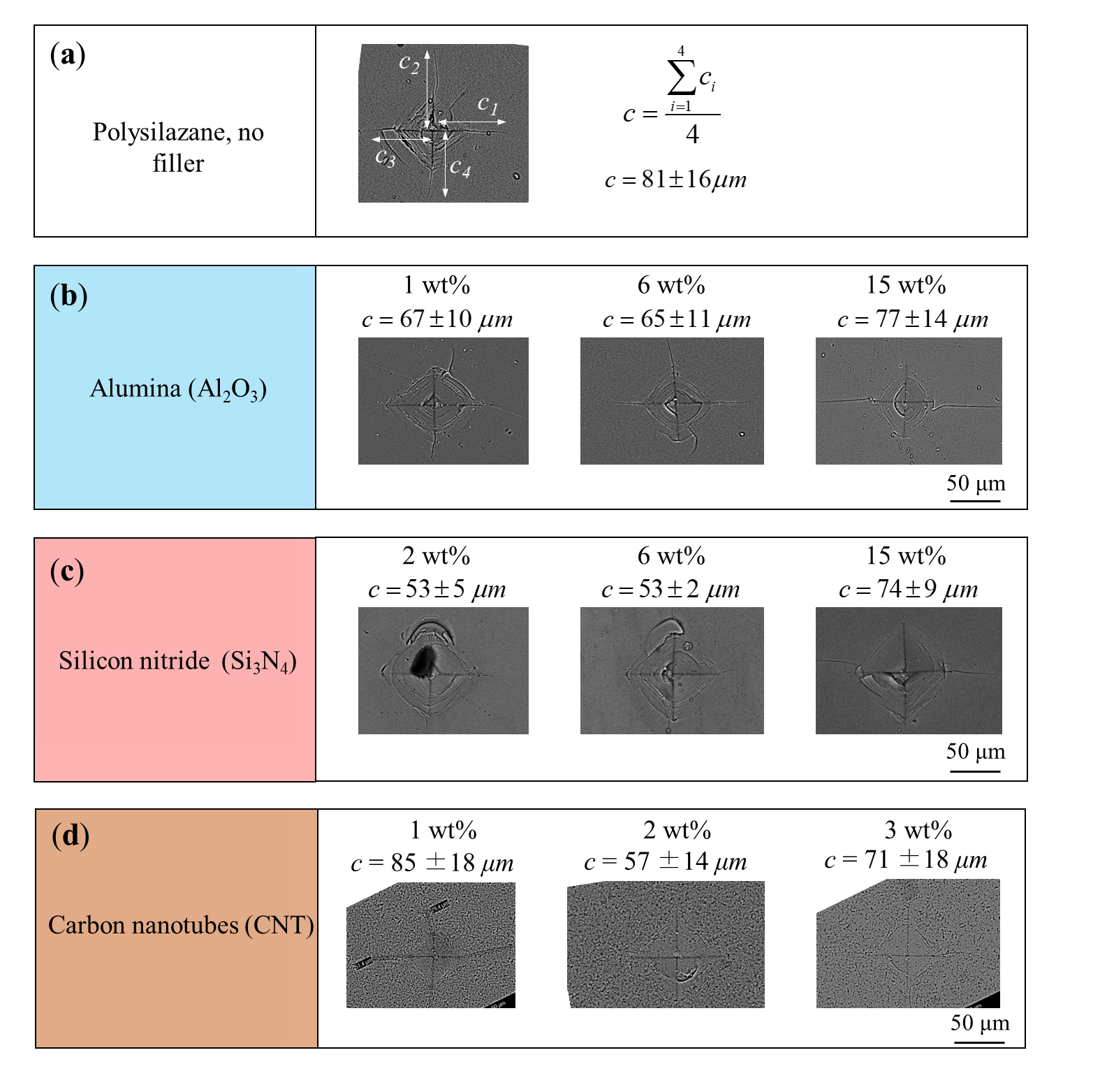


Fig. S1. Representative SEM micrographs of micro-indentation cracks for samples containing nano-fillers. All the cracks were obtained with the same force on PDCs derived from (a) Polysilazane with no filler, and (b-d) Polysilazane with different concentration of (b) Alumina, (c) Silicon nitride, and (d) carbon nanotube nano-fillers.

**SEM-EDS characterization**


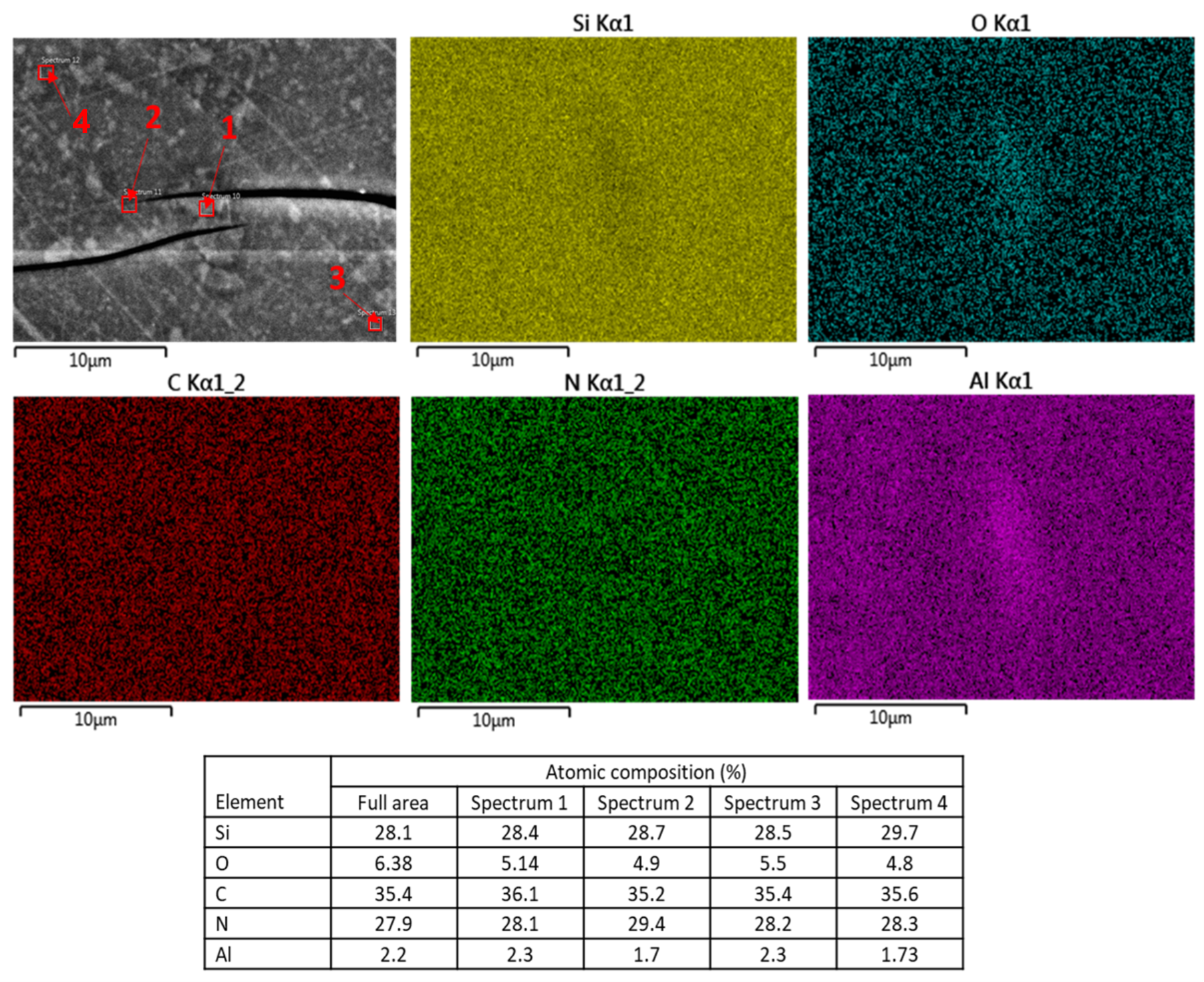


Fig. S2. The EDX elemental mapping of a Al_2_O_3_(6 wt%)-modified PDCs around a crack along with tabulated compositional results showing composition in the bridging region (spectra 1 and 2) in comparison to other locations and the average composition over the full map area.

**Coupled thermogravimetric analysis-Fourier transform infrared spectroscopy (TGA-FTIR)**

As described in the manuscript, TGA (Netzsch STG 449 F1; 10 °C/min heating rate) coupled to an FTIR spectrometer (Bruker Tensor 27) was used to assess the conversion from polymer resin to green to ceramic. Fig. 8 (see main manuscript) showed the evolution of the sample mass during the curing and pyrolysis for the PSZ alone and with 15 wt% of either Si_3_N_4_ or Al_2_O_3_ nanoparticles. In all three cases there was a mass loss beginning ~ 250 °C associated with forming the green state, and a second mass loss during pyrolysis, which peaked just over 600 °C and was complete before reaching 1000 °C. The general shapes of the TGA curves for all three compositions were similar, with only slightly (~2%) less mass loss during pyrolysis for the nanoparticle-filled cases and with slightly more mass loss during curing to form the green (T ≤ 400 °C) for the Si_3_N_4_-filled case. Fig. S2 shows coupled FTIR spectra corresponding to these TGA experiments. The IR spectra indicate the species released during the TGA experiment.


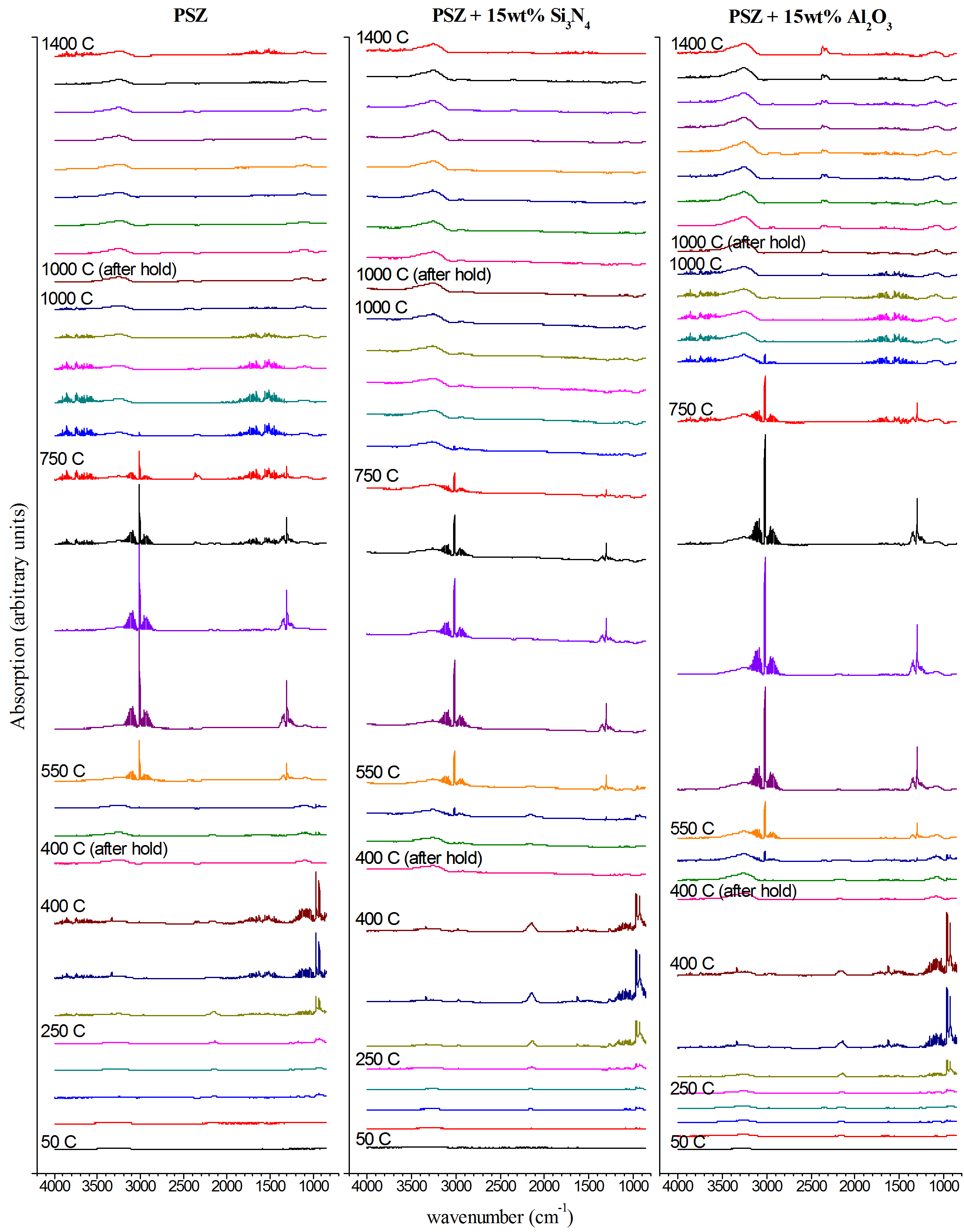


Fig. S3. IR spectra from coupled TGA-FTIR characterization of PSZ and samples with 15 wt% Si_3_N_4_ or Al_2_O_3_ nanoparticle fillers. Spectra, stacked vertically, are shown every 50 °C. Two spectra are shown at T = 400 °C and at T = 1000 °C, with the first spectra taken when initially reaching temperature and the second at the end of the extended holds at these two temperatures.

The main absorption peaks observed during the lower temperature mass loss event (e.g., Fig. S3 at T = 400 °C) correspond to those for NH_3_. Water is also observed, although to a lesser extent for the nanoparticle-filled samples. The primary absorption features observed during the higher temperature event (e.g., Fig. S4 at T = 650 °C) correspond to the spectral features for methane. As described in previous literature [S1], active filler particles were expected to react with hydrocarbons released during pyrolysis. The FTIR shows that methane is available for reaction and that pyrolysis reaches completion by 1000 °C but this does not directly verify a reaction has occurred with the nanoparticles. Considering mass loss during pyrolysis is also reduced and the compositional changes (Table 1, main text), in particular with Si_3_N_4_, the observations collectively suggest the filler is not simply passive. For temperatures between ~700 °C and 1000 °C (e.g., Fig. S5 at T = 850 °C), water is also observed for the PSZ and Al_2_O_3_-containing sample but is not apparent for the Si_3_N_4_-containing sample. This difference could be connected to the superior quality (minimal to no evidence of voids and cracks) in the Si_3_N_4_-containing samples, and also to the compositional results (Table 1, main text), which show much higher oxygen-content for those samples.


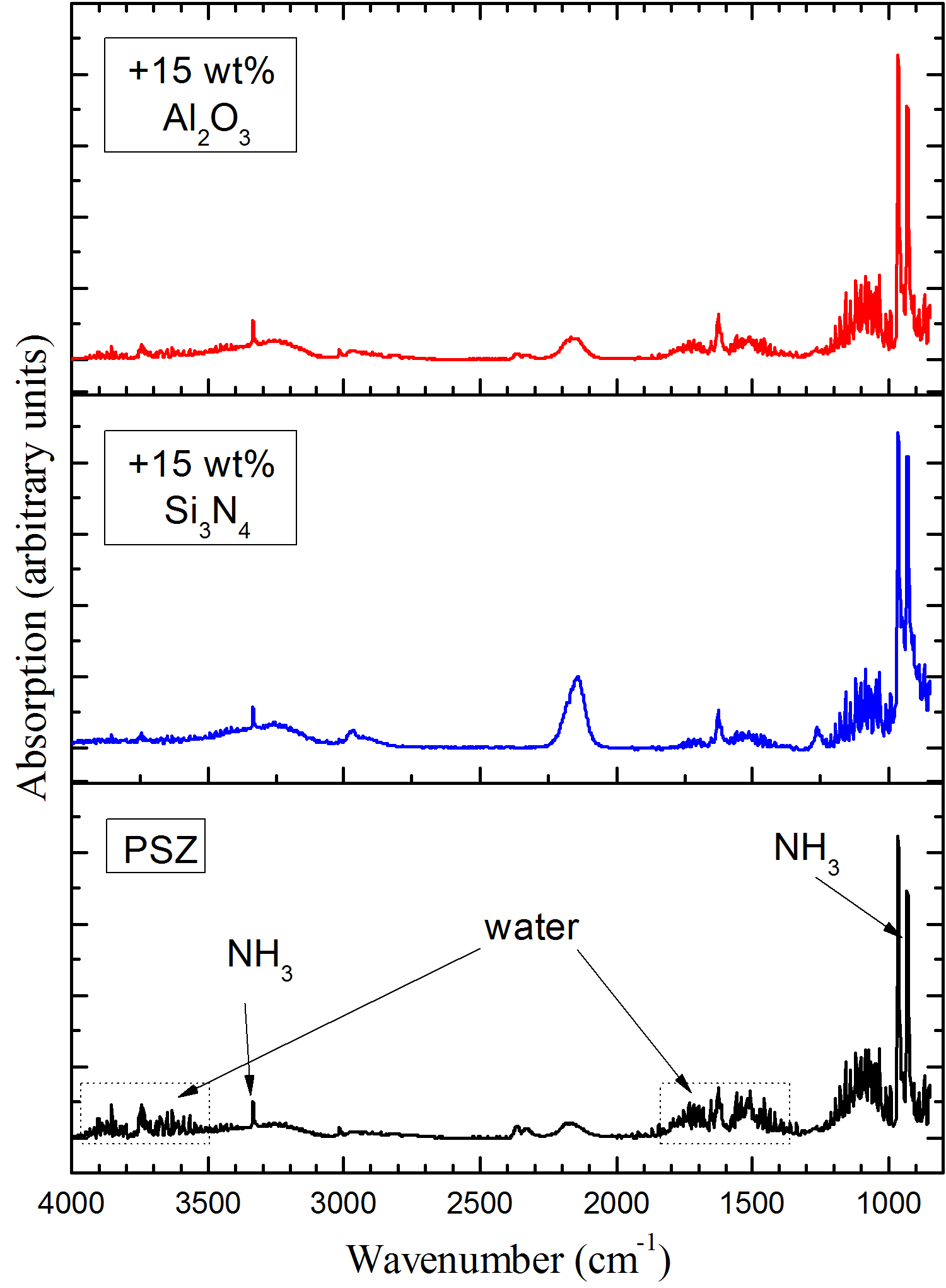


Fig. S4. Coupled FTIR spectra collected at 400 °C during the TGA measurements shown in the main text (Fig. 8).


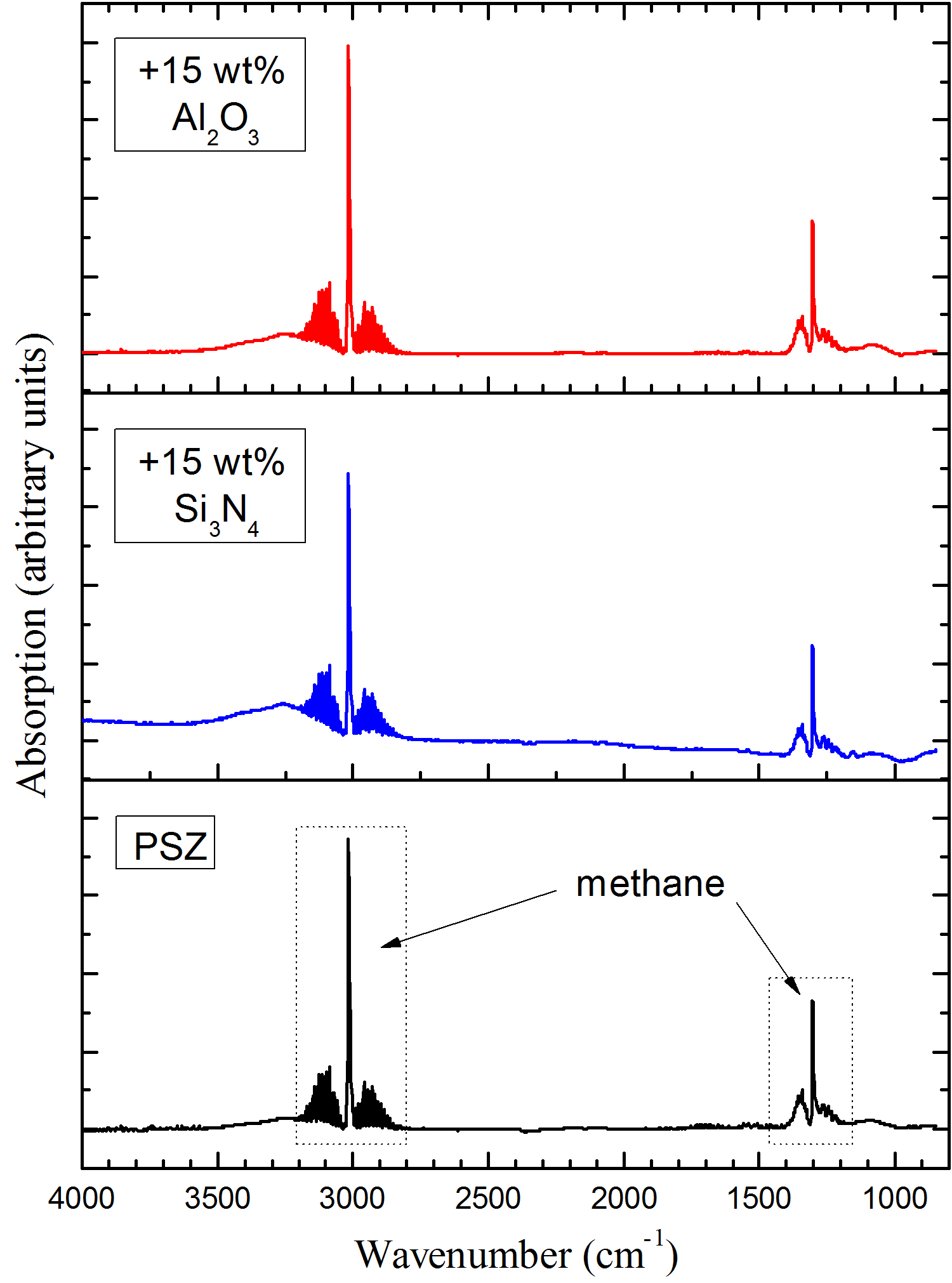


Fig. S5. Coupled FTIR spectra collected at 650 °C during the TGA measurements shown in the main text (Fig. 8).


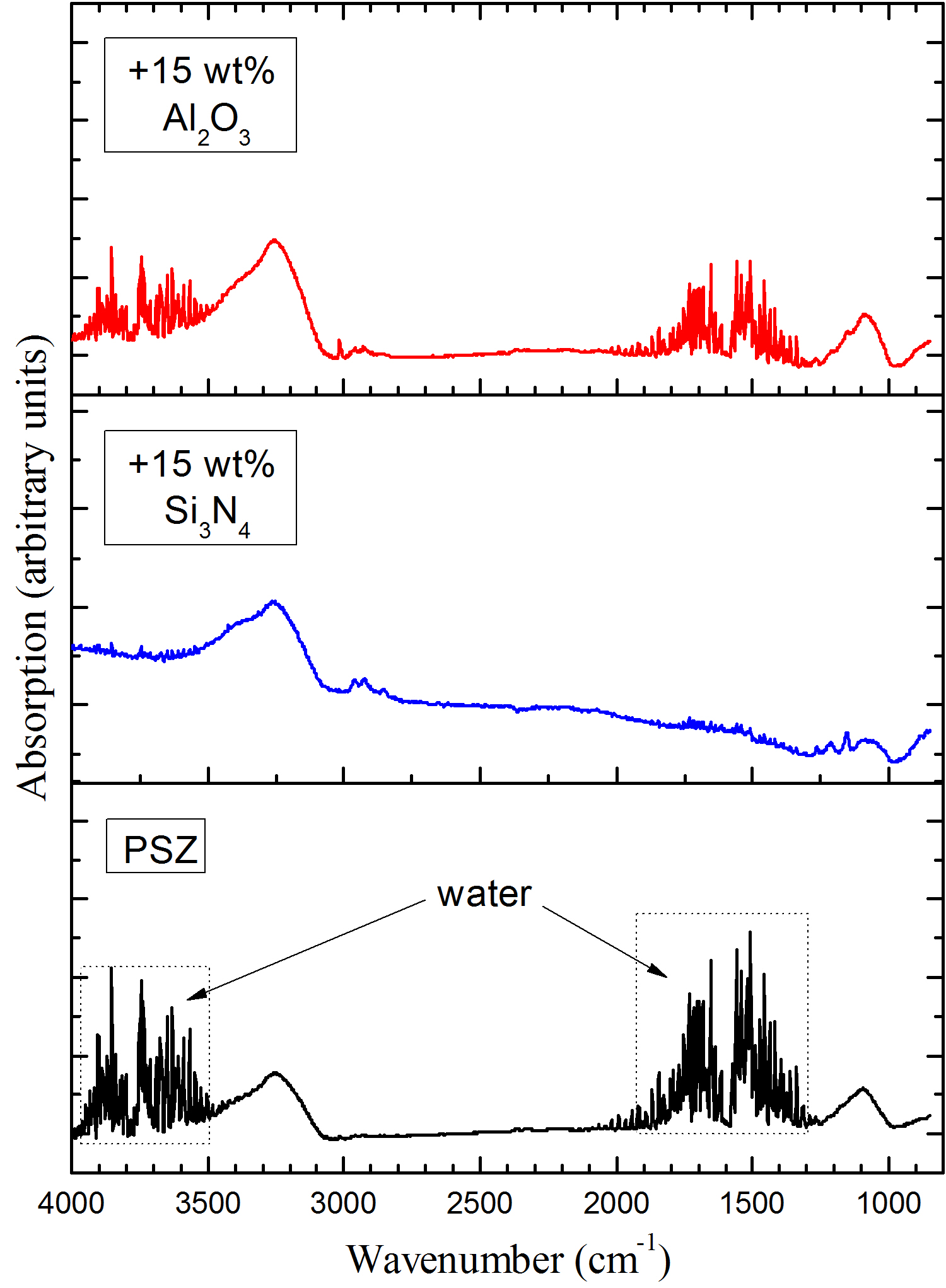


Fig. S6. Coupled FTIR spectra collected at 850 °C during the TGA measurements shown in the main text (Fig. 8).

**X-ray photoelectron spectroscopy (XPS)**

XPS measurements were performed as described in the main text. Atomic composition was determined from survey scans (Figure S6 and Table 1). Survey scans clearly show the characteristic peak for aluminum (as labelled in Figure S6) only for the sample produced with relatively high content of alumina filler. In addition to the compositional changes discussed in the main text, high-resolution XPS spectra (Figure S7) were recorded in order to assess the local chemical environment of Si, O, C and N. The C (1s) spectra shows at least four component peaks, which are assigned to C-Si, C-C, C-O and C-N (in order of increasing binding energy). The x-axis scales of all spectra were calibrated by shifting the C-C peak to a binding energy of 284.8 eV. Due to the overlap of some peaks, the component fitting results (shown in red in Figure S7) are influenced by the fitting method/constraints. These spectra show the local bonding environments are similar across all samples; however, there is a clear shift of the experimental Si (2p) peak to higher binding energy for the samples made with Si_3_N_4_ filler. The component fitting shows that this is due to an increase in Si-O bonding (relative to Si-C) for this sample in comparison to the baseline or other filler cases. This change is consistent with the significant increase in oxygen content for this sample, which is attributed to moisture as supported by the TGA-FTIR and shows the role of the Si3N4 filler in this case is not only the expected reaction with hydrocarbon byproducts. The percentage of C-Si bonding, indicated by the prominence of the low-binding-energy shoulder of the C(1s) peak also appears to decrease with the addition of a filler without silicon. An aluminum peak, assigned to Al-O is observed only for the sample made with added Al2O3 (Figure S8).


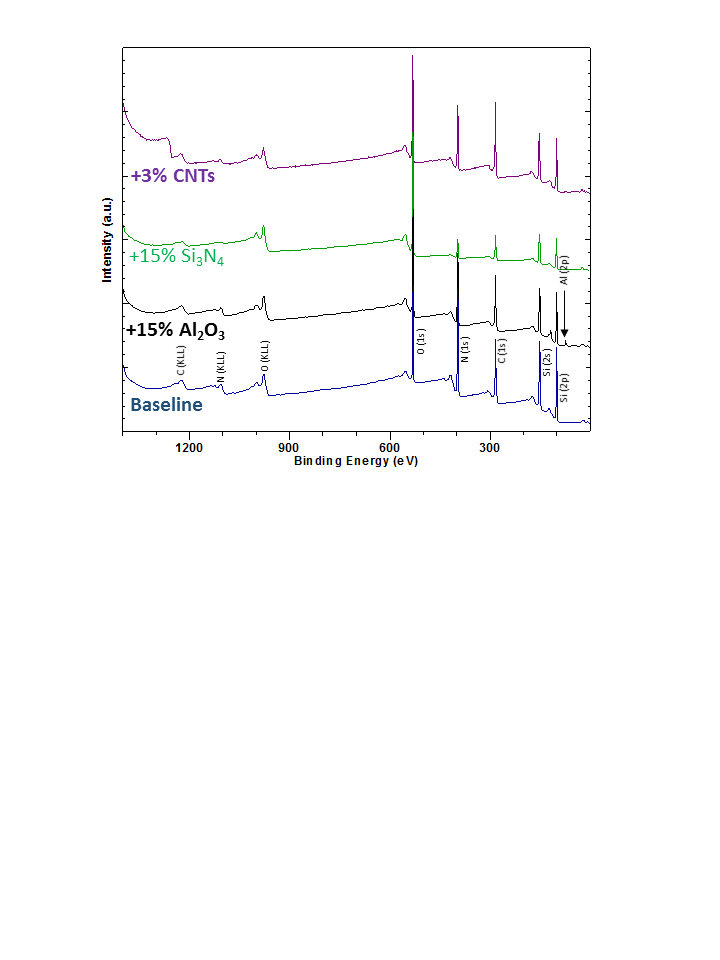


Fig. S7. Representative XPS survey scans for the polymer-derived ceramic samples


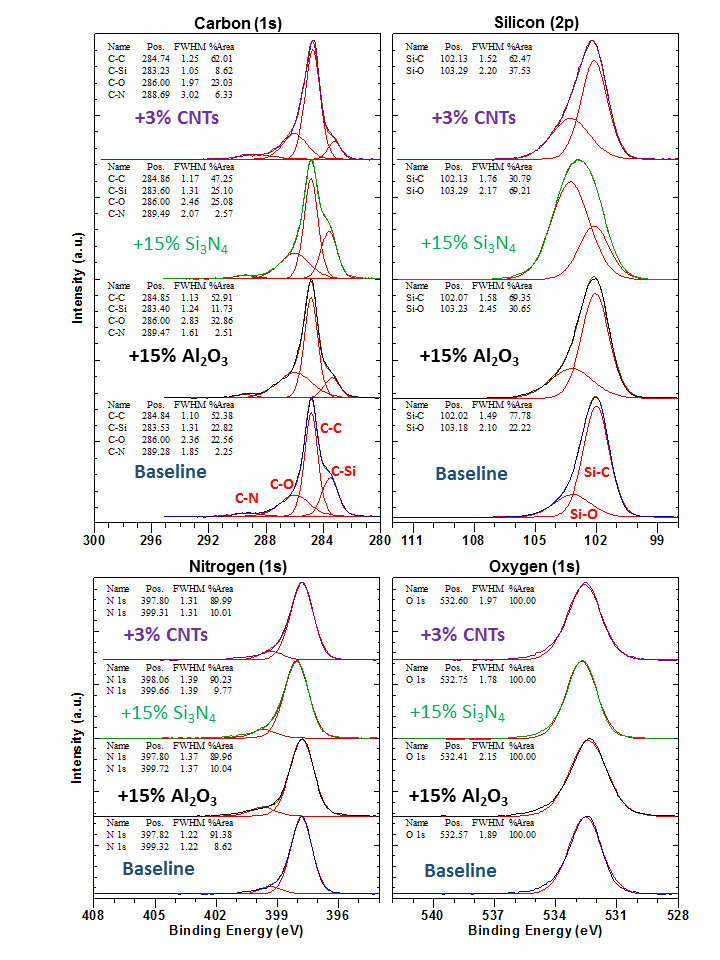


Fig. S8. High-resolution XPS scans (normalized and background-subtracted) with component peak fitting (red) for the C(1s), Si(2p), N (1s) and O (1s) regions showing the **Baseline** ceramic and samples prepared with **15% Al_2_O_3_**, **15% Si_3_N_3_** and 3% CNTs.


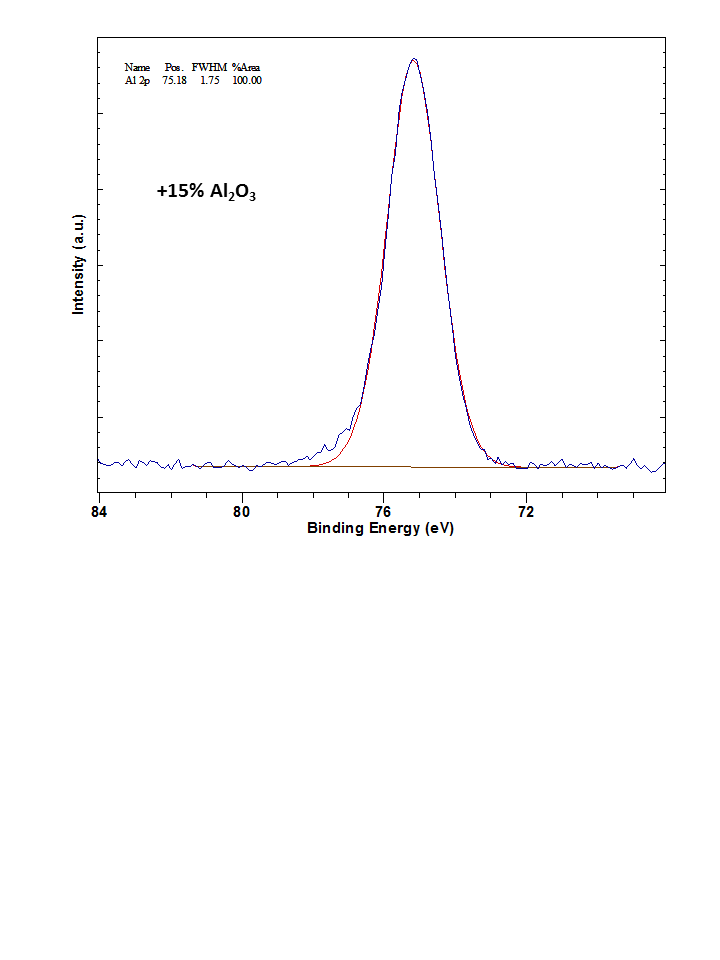


Fig. S9. High-resolution XPS scan in the Al(2p) region for the sample with 15% Al_2_O_3_

[S1] P. Colombo, E. Bernardo, G. Parcianello, Multifunctional advanced ceramics from preceramic polymers and nano-sized active fillers, Journal of the European Ceramic Society, 33 (2013) 453-469.
